# Fibroblasts Influence Metastatic Melanoma Cell Sensitivity to Combined BRAF and MEK Inhibition

**DOI:** 10.3390/cancers13194761

**Published:** 2021-09-23

**Authors:** Delphine Morales, Pascale Vigneron, Ines Ferreira, Warda Hamitou, Mikael Magnano, Laxsika Mahenthiran, Catherine Lok, Muriel Vayssade

**Affiliations:** 1 Université de Technologie de Compiègne, CNRS, Biomechanics and Bioengineering, Centre de Recherche Royallieu—CS 60 319, CEDEX, 60203 Compiègne, France; delphine.morales@outlook.com (D.M.); pascale.vigneron@utc.fr (P.V.); ines.ferreira@etu.utc.fr (I.F.); warda.hamitou@gmail.com (W.H.); mikael.magnano@laposte.net (M.M.); mlaxsika@yahoo.fr (L.M.); 2 Department of Dermatology, CHU Amiens Picardie—Site Nord, CEDEX 1, 80054 Amiens, France; lok.catherine@chu-amiens.fr

**Keywords:** targeted therapy, tissue engineering, 3D human melanoma model, BRAFi + MEKi efficiency, cancer-associated fibroblasts

## Abstract

**Simple Summary:**

Preclinical 3D in vitro coculture models are known to be more complex systems than monolayer cell culture and mimic the physiological environment more closely. Three-dimensional dermal equivalents provide a relevant environment for cutaneous metastatic melanoma cells and are capable of modulating a cancer cell’s response to drugs. We showed that a combined targeted therapy (vemurafenib and cobimetinib) efficiently inhibits cell proliferation and induces apoptosis, especially in the 3D coculture model. A cancer-associated fibroblast population isolated from a cutaneous melanoma was also sensitive to the treatment but with no detectable induction of apoptosis. To better understand the complex crosstalk between melanoma cells and their microenvironment, we compared the influence of conditioned media obtained from healthy or cancer-associated fibroblasts on the response of metastatic melanomas to the drugs. Our data indicate that normal fibroblast supernatants potentialize the therapy’s efficiency, whereas cancer-associated fibroblast secretomes favor melanoma cell survival.

**Abstract:**

The sensitivity of melanoma cells to targeted therapy compounds depends on the tumor microenvironment. Three-dimensional (3D) in vitro coculture systems better reflect the native structural architecture of tissues and are ideal for investigating cellular interactions modulating cell sensitivity to drugs. Metastatic melanoma (MM) cells (SK-MEL-28 BRAF V600E mutant and SK-MEL-2 *BRAF* wt) were cultured as a monolayer (2D) or cocultured on 3D dermal equivalents (with fibroblasts) and treated with a BRAFi (vemurafenib) combined with a MEK inhibitor (MEKi, cobimetinib). The drug combination efficiently inhibited 2D and 3D MM cell proliferation and survival regardless of their BRAF status. Two-dimensional and three-dimensional cancer-associated fibroblasts (CAFs), isolated from a cutaneous MM biopsy, were also sensitive to the targeted therapy. Conditioned media obtained from healthy dermal fibroblasts or CAFs modulated the MM cell’s response differently to the treatment: while supernatants from healthy fibroblasts potentialized the efficiency of drugs on MM, those from CAFs tended to increase cell survival. Our data indicate that the secretory profiles of fibroblasts influence MM sensitivity to the combined vemurafenib and cobimetinib treatment and highlight the need for 3D in vitro cocultures representing the complex crosstalk between melanoma and CAFs during preclinical studies of drugs.

## 1. Introduction

The first-line therapy for patients with metastatic melanoma (MM) with a BRAF V600E mutation is a BRAF inhibitor (BRAFi) such as vemurafenib [1]. It has now been shown that the therapeutic action of BRAFi is improved when the compound is used in combination with an MEK inhibitor (MEKi) [2,3,4]. These therapies are based on thorough knowledge and study of the molecular mechanisms involved in the development of melanomas and, thus, make possible a truly personalized approach to patient care. However, some patients with the BRAF mutation either do not respond to treatment (approximately 10 to 20%) [5,6,7] or develop resistance, very often within a median time of 6 months after starting treatment [8]. Therapeutic failures are not only linked to these resistance phenomena but also to the significant side effects of these compounds.

Cancer cell sensitivity to drugs is highly dependent on the microenvironment (cell–cell and cell–extracellular matrix interactions, paracrine signaling, etc.) [9]. Preclinical 3D in vitro coculture models are known to be more complex systems than monolayer cell cultures and mimic the physiological environment more closely. Three-dimensional dermal equivalents provide a relevant environment for metastatic melanoma cells and are capable of modulating a cancer cell’s response to drugs [10,11]. We recently developed a 3D human dermal equivalent (dermal fibroblasts embedded in a collagen gel) to mimic the cutaneous environment for MM cells (SK-MEL-28 BRAF V600E mutant and SK-MEL-2 BRAF wt, NRAS Q61R). This model made it possible to observe the effective inhibition of BRAFi on the proliferation of MM cells (wild-type or mutated BRAF V600E) in coculture within the 3D models: SK-MEL-2 cells were sensitive to BRAFi in the 3D coculture, while they were resistant in the 2D configuration, highlighting that paracrine signaling in coculture models and extracellular matrix components are key points for drug efficiency. This shows the value of studying MM sensitivity to targeted first-line therapy treatment, the combination BRAFi + MEKi.

Cancer-associated fibroblasts (CAFs) are activated fibroblasts with mesenchymal cell lineage and heterogeneity in the tumor microenvironment [12]. In MM, CAFs express high levels of the mesenchymal protein vimentin, PDGFR, or α-SMA, and they secrete soluble mediators (such as pro-inflammatory proteins IL-6, IL-8, HGF, etc.) [13,14]. They are the most abundant stromal cells in the cutaneous MM environment and have a major influence on melanoma growth and therapy resistance [13,14], especially to BRAFi [15].

In the present study, we analyzed the response of MM cell lines (BRAF mutant and BRAF wild-type) cultured as a monolayer or within a dermal equivalent to a drug combination, BRAFi + MEKi. We also isolated a CAF population from a patient with cutaneous MM and we compared the influence of conditioned media obtained from healthy fibroblasts or CAFs on MM cells’ response to the drugs.

## 2. Materials and Methods

### 2.1. Cell and Tissue Models

#### 2.1.1. Culture Conditions of Established Cell Lines

The human metastatic melanoma (MM) cell lines (SK-MEL-28 ATCC^®^ HTB-72^TM^ and SK-MEL-2 ATCC^®^ HTB-68^TM^) were obtained from the American Type Culture Collection. Human dermal fibroblasts, neonatal (HDFn) were purchased from Life Technologies (Cascade biologics, Invitrogen). Cells were cultured as monolayers in MEM (SK-MEL-28, SK-MEL-2) or DMEM (HDFn). The cell culture media (Gibco, Invitrogen, Villebon sur Yvette France) were supplemented with 10% fetal bovine serum (FBS, Gibco, Invitrogen for melanoma cells; HyClone^TM^ (U.S) characterized for dermal fibroblasts), 2 mM L-glutamine (Gibco, Invitrogen), penicillin (100 U/mL, Gibco, Invitrogen) and streptomycin (100 µg/mL, Gibco, Invitrogen). All cell lines were maintained at 37 °C in an air atmosphere with 5% CO_2_. Melanoma cell lines were used between passages 25 and 40 and HDFn between passages 3 and 8.

#### 2.1.2. Isolation and Culture of MM Cancer-Associated Fibroblasts (CAFs)

**Figure A1:**
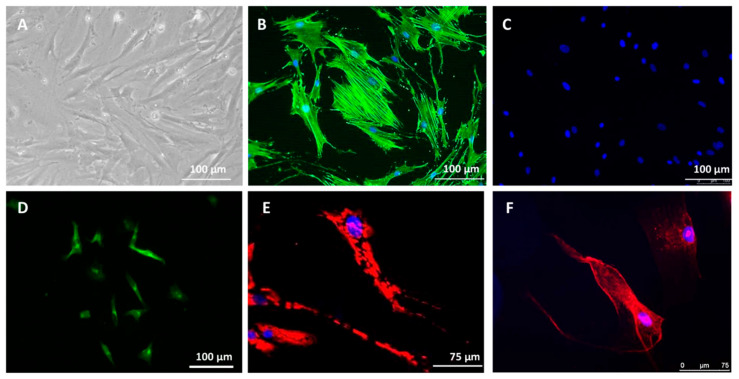
CAF characterization. (**A**) Observation of CAF morphology; (**B**) F-actin staining using phalloidin-FITC (*FP AZ0130, Fluoprobes*) in isolated CAFs. Fibroblast nuclei were stained blue with 4′,6-diamidino-2-phenylindole); (**C**) CAFs were negative for Melan-A expression; (**D**) SK-MEL-2 cells were used as positive control for Mela-A expression; (**E**) CAFs strongly expressed vimentin protein (red staining); (**F**) CAFs expressed the PDGFRβ marker (red staining).

CAFs were isolated from a patient’s biopsy of cutaneous melanoma metastases (BRAF wt, NRAS Q61R) after informed consent according to ethical approval “CPP Nord-Ouest, France, code: PI2016_843_0021” and following the Declaration of Helsinki on human rights. In brief, the tumoral tissue was divided into small pieces and digested with 2.4 U/mL dispase (Roche, Basel, Switzerland), followed by digestion with 0.3 mg/mL type I collagenase (Sigma, Saint-Quentin-Fallavier, France), so that the final suspension consisted of separated cells. CAFs were selected by short trypsinization of the dissociated cells and the selective adherence method [16]. If nests of melanoma cells grew visibly on top of the fibroblasts, the cell cultures were rinsed twice with PBS and briefly trypsinized (approximately 1 min at room temperature) to detach only the cells on the top layer, allowing the CAFs to remain adherent. Isolated CAFs were cultured in DMEM supplemented with 10% FBS (Hyclone), 2 mM L-glutamine, penicillin (100 U/mL), and streptomycin (100 µg/mL). The cells were maintained at 37 °C in an air atmosphere with 5% CO_2_. Isolated CAFs were analyzed for their morphology and the expression of markers (Figure A1). For experiments, CAFs were used under passage 5.

#### 2.1.3. Monolayer Cell (2D) Treatment

For treatment, MM cells and fibroblasts (HDFn and CAFs) were seeded on Falcon^®^ polystyrene 24-well plates (10^4^ cells/cm^2^) for 24 h allowing cells to adhere and spread. Cells were then treated with increasing concentrations of MEKi (from 0.2 µM to 10 µM, cobimetinib, Cotellic^®^, Cayman Chemical; Ann Arbor, MI, USA) and 2 µM BRAFi (vemurafenib (PLX4032), Zelboraf^®^, Cayman Chemical) for 48 h. BRAFi and MEKi were dissolved in DMSO (stock concentration 10 mM) and untreated conditions corresponding to cells treated with 0.001% DMSO (*v/v*).

#### 2.1.4. Dermal Equivalent Model and 3D Coculture Treatment

To obtain the dermal equivalent, the original bovine collagen type I solution (Cell systems Bio) was diluted from 3 mg/mL to 1 mg/mL by adding MEM 10X (Life Technologies, Villebon sur Yvette, France), 1% L-glutamine (200 mM), 10% FBS (HyClone^TM^), and sterile water. The mixed solution was neutralized by NaHCO_3_ (1 N) and the color change of the phenol red from yellow to red-orange in solution was used to monitor the pH (~7.2). HDFn or CAF cells (5.10^4^ cells/insert) were then added to the neutral collagen solution. Three mL of this solution was subsequently added to each of the 6 inserts in a Nunc™ culture plate (Thermo Fisher Scientific, Courtaboeuf, France) and incubated at 37 °C to obtain cells embedded within the collagen scaffold. Culture medium (complete DMEM, 3 mL) consisting of 90% (*v/v*) DMEM, 10% (*v/v*) FBS (HyClone™), 1% HEPES, 2 mM L-glutamine, 50 U/mL penicillin and 50 µg/mL streptomycin was then added after 2 h to each well of the Nunc™ culture plate for cell culture. Three-dimensional dermal equivalent models were incubated at 37 °C with 5% CO_2_ (medium was changed every 2 or 3 days) for 8 days and treated with BRAFi and MEKi for 48 h.

For coculture experiments, MM lines (10^4^ cells) were added to the top of the HDFn dermal model (day 7) with 1 mL of cell culture medium and cultured for 24 h before BRAFi and MEKi treatment for 48 h. The same timeframe for assays and drug exposition was used on both the 2D and 3D coculture models to make a valid comparison possible.

#### 2.1.5. Conditioned Media Preparation

HDFn or CAFs were seeded and cultured as a monolayer or in a 3D collagen gel as described above with complete DMEM. After 48 h of treatment with BRAFi + MEKi, the conditioned media (CM) were collected, centrifuged (500× *g*, 5 min), the supernatant added to 1:1 with fresh complete MEM (+ BRAFi + MEKi), and placed on SK-MEL-2 cells for 48 h to assess the paracrine effect of healthy or cancer-associated fibroblasts on MM cells’ response.

### 2.2. Cell Proliferation Assays

#### 2.2.1. Cell Metabolic Activity Assay

Metabolic activity was quantified using MTS assay (Cell Titer 96^®^ Aqueous One Solution, Promega^®^, Charbonnières-les-Bains, France). MTS solution (200 μL) was added to 1 mL of culture medium in each well for 2D cultures and 5 mL of culture medium in each well for 3D coculture models. After 2 h of incubation at 37 °C, 3 aliquots (100 μL) from each well were transferred into a 96-well cell culture plate (Corning Life Science, Samois sur Seine France). Optical density (OD) of samples was read using a Bio-Rad Model 680 microplate reader at 490 nm. The blank consisted of the culture medium containing MTS that was not bioreduced by cells. The absorbance reading of the blank was subtracted from all samples. Optical densities from test samples were then divided by the value obtained for the control and the metabolic activity was expressed as percentages. All experiments were performed at least twice in triplicate (*n* ≥ 6). IC50 values were calculated using non-regular regression (sigmoidal dose response) of the plot of percentage inhibition vs. inhibitor concentration with GraphPad Prism (Graph Pad Software, San Diego, CA, USA) and Regressi software (Softonic, Barcelona, Spain).

#### 2.2.2. EdU Labelling

EdU staining (Click-iT^®^ EdU Alexa Fluor^®^ 488 imaging kit, Life Technologies) was conducted to measure cells’ ability to proliferate with BRAFi and MEKi treatment (2D and 3D cultures). After 48 h of exposure to the treatment, samples were first incubated in culture medium containing 10 μM EdU for 10 h to allow the incorporation of EdU into the DNA of proliferating cells. 2D cultured cells were then fixed for 15 min with 4% formaldehyde and permeabilized with 0.5% Triton X-100 for 20 min. A Click-iT^®^ reaction cocktail (100 μL) prepared from the assay kit was added. Samples were incubated at room temperature for 30 min and protected from light. 4′,6-diamidino-2-phenylindole (DAPI) solution (1 µg/mL) was applied to each sample and incubated at room temperature for 30 min. Slides were mounted in Vectashield H-1000 (Vector laboratories, Les Ulysses, France) and examined with epifluorescence microscopy (Leica DMI6000, Nanterre, France, exciting light at 450–490 nm and 512–542 nm). For 3D coculture models, frozen sections of samples were prepared: samples were immersed in 1 M sucrose for 2 h then 2 M sucrose for 2 h at 4 °C to displace the water from cellular spaces. Samples were then blotted to remove excess sucrose solution and embedded in OCT compound through a flash freezing procedure (samples were immersed in OCT inside a cylinder mold and placed upon the upper surface of liquid nitrogen for about 5 min). Flash-frozen tissue sections with a thickness of 5 μm were then cut onto Superfrost^TM^ Ultra Plus slides by Cryostat LEICA CM3050 S (France). Tissues on slides were fixed (3.7% formaldehyde) and permeabilized (0.5% Triton^®^ X-100). EdU incorporation and DAPI staining were detected as described above. All experiments were performed at least twice and EdU positive/negative cells were counted (*n* ≥ 6): for the 2D models 700 total cells for each condition were counted and for the 3D models at least 200 total cells.

#### 2.2.3. Cell Proliferative Index

After BRAFi and MEKi treatment of the CAF dermal equivalent model, the culture medium was removed and the collagen gel containing cells was digested by 3 mL HBSS (Life Technologies) containing 200 U/mL type I collagenase (Life Technologies) for 5 h at 37 °C. Dissociated cells were then collected and counted using Malassez hemocytometer after Trypan blue staining. The cell proliferative index was calculated as the ratio between the number of living cells after treatment and the number of untreated cells. Cell viability was determined as the ratio between living cells and the number of total cells. All experiments were performed twice in triplicate (*n* = 6).

### 2.3. Histological Observation of the 3D Coculture Models 

The 3D coculture samples were immersed in 4% paraformaldehyde overnight at 4 °C and dehydrated using a succession of ethanol baths and one xylene bath (1 h each). Each sample was embedded in paraffin blocks and paraffin sections of 7 µm were then cut with a microtome (LEICA 2245). The cell organization in the reconstructed tissue was morphologically characterized using hematoxylin, eosin and saffron (RAL Diagnostics and Q Path; Martillac, France) stained paraffin sections. Images were taken with a LEICA DMC 4500 Camera through a LEICA DM 1000 LED microscope (France).

### 2.4. Apoptosis Detection

In order to detect DNA fragmentation, a Click-iT^®^ Plus assay Alexa Fluor 594 (Life Technologies) was used. After BRAFi and MEKi treatment, 2D cell cultures were fixed for 15 min with 4% formaldehyde and permeabilized with 0.25% Triton X100 for 20 min. For dermal equivalent models, samples were immersed in 4% formaldehyde overnight at 4 °C and dehydrated. Each sample was then embedded in paraffin blocks. Paraffin sections of 7 µm were cut with a microtome (LEICA 2245) and deparaffinization of tissue sections was necessary before the TdT reaction (succession of baths: xylene 5 min, 100% ethanol 5 min, 95% ethanol 5 min, 70% ethanol 5 min, and 1X PBS 5 min). Tissue sections were permeabilized with the permeabilization reagent (Proteinase K solution) provided in the kit for 15 min. For the positive control, cells were incubated with 1 unit of DNase I (Sigma) diluted in 1X DNase I reaction buffer (20 mM Tris HCl, pH 8.4, 2 mM MgCl_2_, 50 mM KCl) for 30 min at room temperature.

In total, 100 µL TdT reaction buffer was added to each coverslip for 10 min at 37 °C. A Click-iT^®^ Plus TUNEL reaction cocktail was then added for 30 min at 37 °C protected from light. Slides were mounted in Vectashield H-1000 (Vector laboratories) and examined using epifluorescence microscopy (LEICA DMI 6000, exciting light at 450–495 nm and at 620–750 nm). Positive and negative cells for the TUNEL staining were counted to establish percentages of apoptotic cells: for 2D models 700 total cells for each condition were counted and for the 3D models at least 200 total cells.

### 2.5. Statistical Analysis

All statistical evaluations were performed using GraphPad InStat software. Continuous variables are expressed as means ± standard deviation. As most of the continuous values measured had a non-Gaussian distribution, the non-parametric Kruskal–Wallis test followed by Dunn post-hoc test was used to compare more than two independent groups, and the non-parametric Mann–Whitney test was used to compare two independent groups. A value of *p* < 0.05 was taken as significant. 

## 3. Results

The response of metastatic melanoma (MM) cells (SK-MEL-28, SK-MEL-2) and human dermal fibroblasts (HDFn, CAFs) to BRAFi and MEKi was analyzed (2D and 3D cultures). All cells and reconstructed tissues were treated with increasing concentrations of MEKi (from 0.2 to 10 µM) combined with BRAFi (2 µM).

### 3.1. The Drug Combination Strongly Inhibits the Proliferation of MM Cell Lines Cultured as Monolayer Regardless of Their BRAF Status

**Figure 1:**
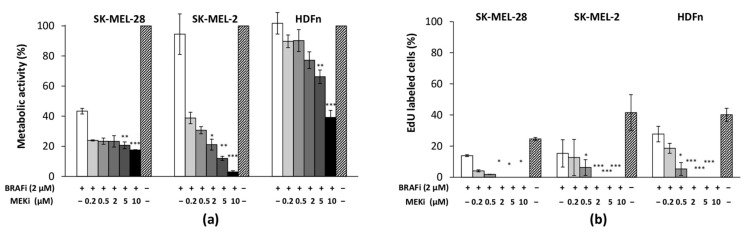
Characterization of the effect of BRAFi and MEKi on MM cells and healthy fibroblasts cultured as a monolayer. Melanoma cells (SK-MEL-28, SK-MEL-2) and human dermal fibroblasts (HDFn) were treated with vemurafenib (2 µM) combined with different concentrations of cobimetinib for 48 h. Cell proliferation relative to untreated controls was assessed by means of (**a**) cell metabolic activity (MTS assay) and (**b**) DNA synthesis (EdU assay). Results are means ±  s.d. of three experiments performed in triplicate: * *p* < 0.05; ** *p* < 0.01; *** *p* < 0.001 indicate significant difference vs. untreated cells.

**Figure A2:**
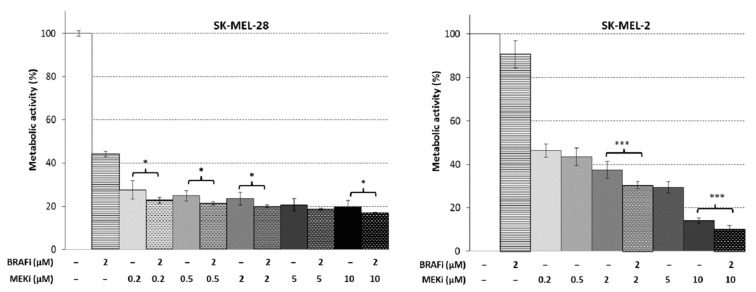
SK-MEL-28 and SK-MEL-2 cell response to combined BRAFi + MEKi or to MEKi single treatment (from 0.2 to 10 µM). Cells were cultured as a monolayer, treated with vemurafenib + cobimetinib or cobimetinib alone during 48 h, and metabolic activity was quantified using MTS assay. *n* = 6; * *p* < 0.05 and *** *p* < 0.001 indicate significant difference between cells treated with BRAFi + MEKi vs. cells treated with MEKi alone (Mann–Whitney test).

The effect of drug association on cell proliferation capacity was assessed using different methods. MTS assay showed a significant decrease in the metabolic activities of MM cells and healthy fibroblasts (HDFn) cultured for 48 h with BRAFi + MEKi (Figure 1a). BRAFi (2 µM) treatment alone strongly decreased SK-MEL-28 metabolic activity by about 60% and the association with MEKi made it possible to reach 80% cell proliferation inhibition whatever concentration of MEKi was used. For the SK-MEL-2 cells, data revealed no effect of BRAFi alone, but the drug combination was particularly efficient for inhibiting cell proliferation with a MEKi dose-dependent effect: SK-MEL-2 metabolic activity was found to be 95% lower than for untreated cells. The drug association significantly inhibited the MM cell proliferation much more strongly than MEKi alone, suggesting that BRAF inhibition was not redundant to MEK inhibition (Figure A2). HDFn cells were not sensitive to 2 µM BRAFi, but their metabolic activity significantly decreased with combined therapy (up to 60%). Overall cell viabilities (estimated by cell counting and trypan blue exclusion assay) remained high (90%) except for the highest MEKi concentration (10 µM) where values were found below 70% [17]. The percentages of proliferating cells were determined after EdU incorporation in treated cells (Figure 1b). Again, a significant and dose-dependent decrease in DNA synthesis was observed when MM and HDFn cells were cultured with the drug combination: the inhibitory effect of the compounds became significant from 0.5 µM or 2 µM MEKi associated with 2 µM BRAFi.

### 3.2. The Drug Combination Inhibits the Proliferation of MM Cells Cocultured on Dermal Equivalents (HDFn)

**Figure 2:**
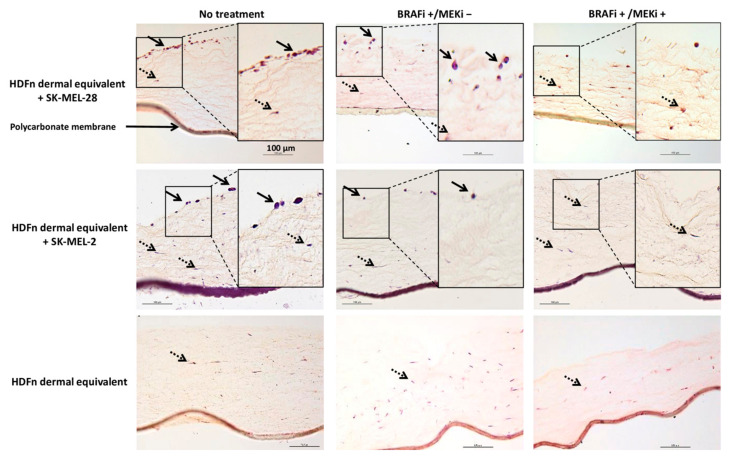
Histological images of 3D coculture models upon BRAFi and MEKi treatment. Dermal equivalents were obtained by seeding HDFn cells in a type I collagen gel. Fibroblasts were cultured in 3D for 7 days. Melanoma cells (SK-MEL-28 or SK-MEL-2) were then added for 24 h. The coculture models were treated with 2 µM of BRAFi and 2 µM of MEKi for 48 h and stained with hematoxylin-eosin-saffron. The melanoma cells on the dermal equivalent are indicated with black arrows. Fibroblasts (dashed arrows) were found inside the tissue and aligned with the collagen fibers. Inserts show a higher magnification of the pictures. Scale bar = 100 µm (200×).

Dermal equivalents were established to mimic the microenvironment of cutaneous MM as previously described [11]. Briefly, the dermal equivalents were obtained by culturing HDFn in a collagen gel for 7 days. MM cells were added and cultured on the reconstructed tissue for 3 days (48 h treatment with inhibitors), then, cell response was characterized histologically (Figure 2): in the untreated cocultures, MM cells were found on the top of the reconstructed tissue. When treated with 2 µM BRAFi alone (BRAFi+/MEKi−), a decrease in the number of SK-MEL-28 and SK-MEL-2 cells was observed, but few cancerous cells remained on the dermal equivalent (black arrows). No MM cells were observed when the cocultures were treated with the inhibitor combination (2 µM BRAFi + 2 µM MEKi). 

**Figure 3:**
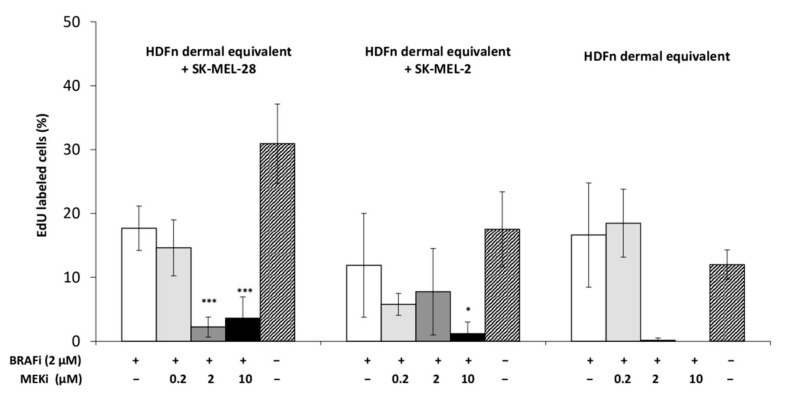
Effect of BRAFi and MEKi on MM cell proliferation in the 3D HDFn dermal equivalent. Coculture models were treated with 2 µM of BRAFi and different concentrations of MEKi for 48 h. Total cell proliferation (MM + fibroblasts) relative to untreated controls was assessed by means of DNA synthesis (EdU assay). Results are means ±  s.d. of three experiments carried out in triplicate: * *p* < 0.05; *** *p* < 0.001 indicate significant difference vs. untreated cells.

**Figure A3:**
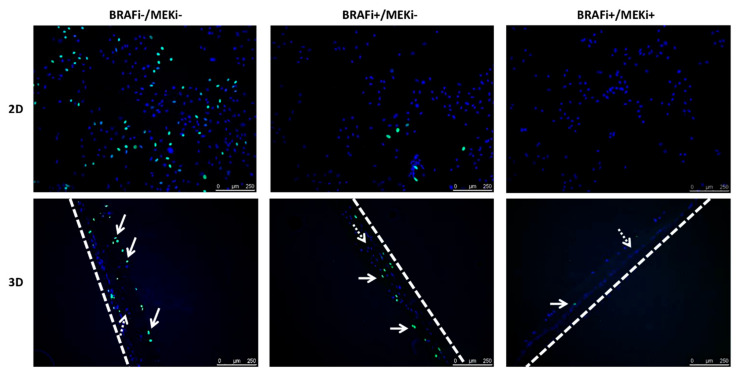
EdU-labeled SK-MEL-28 cells cultured as a monolayer (2D) or in the dermal equivalent (3D) during BRAFi (2 µM) and MEKi treatment (2 µM).For the 3D coculture model, EdU positive melanoma cells are indicated with white arrows and fibroblasts with dashed arrows. The polycarbonate membranes from the inserts are indicated with dashed lines. Scale bar = 250 µm.

The inhibitory effect of the combined compounds was quantified on the 3D cocultures with an EdU assay (Figure 3, Figure A3). Incorporating and labelling the EdU in the proliferating cells revealed the significant effect of BRAFi + MEKi on MM cells; proliferation of both cell lines (SK-MEL-28 and SK-MEL-2) cultured on dermal equivalents decreased during treatment. BRAFi alone (2 µM) decreased SK-MEL-28 and SK-MEL-2 cell proliferation but was inefficient on HDFn cells; when MEKi was added, all cell proliferation decreased significantly. Overall cell viabilities also decreased in coculture models to reach 70% when reconstructed tissues were treated with the highest concentration (2 µM BRAFi + 10 µM MEKi) [17]. 

### 3.3. The Drug Combination Is Cytotoxic for MM Cells Cultured as a Monolayer or in a 3D Coculture Model

**Figure A4:**
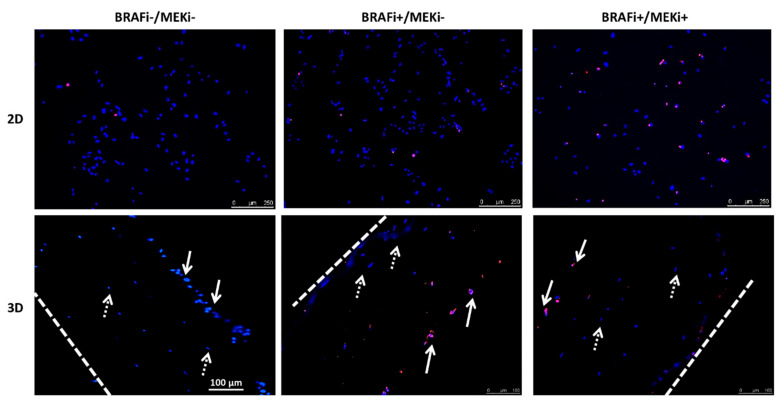
TUNEL-labeled SK-MEL-2 cells cultured as a monolayer (2D) or in the dermal equivalent (3D) during BRAFi (2 µM) and MEKi treatment (2 µM). For the 3D coculture model, TUNEL positive melanoma cells are indicated with white arrows and fibroblasts with dashed arrows. The polycarbonate membranes from the inserts are indicated with dashed lines. Scale bar = 250 µm (2D) or 100 µm (3D).

As the cell viabilities clearly decreased when the 2D and 3D models were treated with BRAFi and MEKi, we investigated the presence of apoptosis in cells (TUNEL assay) (Table 1, Figure A4). Our data indicate that the BRAFi treatment induced low DNA fragmentation in MM and HDFn cells in the 2D and 3D configurations, while the combined treatment clearly induced apoptosis for the different cell types. Importantly, the highest levels of apoptotic nuclei were observed in the coculture models (MM cells on the dermal equivalent) (Table 1). 

### 3.4. The Drug Combination Inhibits the Proliferation of Cancer-Associated Fibroblasts

Cancer-associated fibroblasts (CAFs) were isolated from a biopsy of cutaneous melanoma metastases (patient diagnosed with MM with an NRAS mutation (Q61R) but a BRAF wild-type). CAFs were spindle-shaped and flattened cells and they expressed mesenchymal markers; CAFs were found to be strongly positive for vimentin and PDGFRβ expression and negative for Melan-A, as expected (Figure A1).

**Figure 4:**
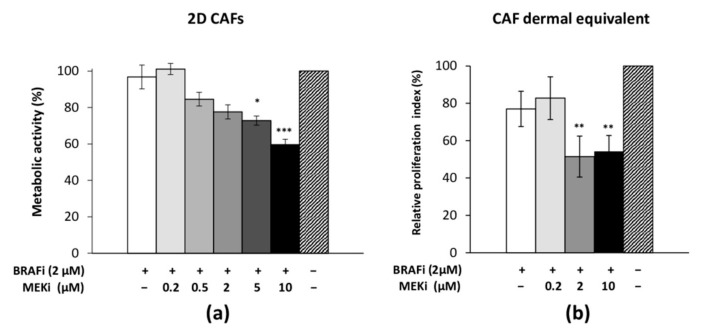
Characterization of the effect of BRAFi and MEKi on cancer-associated fibroblasts (CAFs) cultured as a monolayer or as dermal equivalents. CAFs were treated with BRAFi (2 µM) combined with different concentrations of MEKi for 48 h. Cell proliferation relative to untreated controls was assessed by means of (**a**) cell metabolic activity (MTS assay) and (**b**) cell counting. Results are means ± s.d. of three experiments carried out in triplicate: * *p* < 0.05; ** *p* < 0.01; *** *p* < 0.001 indicate significant difference vs. untreated cells.

The CAF response to the drug combination was investigated when cells were organized in a monolayer or a dermal equivalent (Figure 4). Two-dimensional CAFs did not modify their metabolic activity when they were treated with BRAFi alone or with low concentrations of the combined compounds (2 µM BRAFi + 2 µM MEKi) (Figure 4a). With increasing concentrations of MEKi (5 or 10 µM), a significant decrease in CAF metabolic activity was observed. CAFs were successfully cultured in collagen I gel; in this 3D configuration and on treatment with 2 µM BRAFi, a 20% decrease in the number of cells compared to the untreated condition was observed (Figure 4b). When increasing MEKi concentrations (from 0.2 to 10 µM) were added to 2 µM BRAFi, a significant inhibitory effect (about 50%) was detected for concentrations greater than or equal to 2 µM of MEKi. The viability of CAFs was not affected by the treatment, either in combination or not, and no DNA fragmentation was observed [18]. 

### 3.5. The Microenvironment (Healthy Fibroblasts vs. CAFs) Influences MM Sensitivity to the Drug Combination

**Figure 5:**
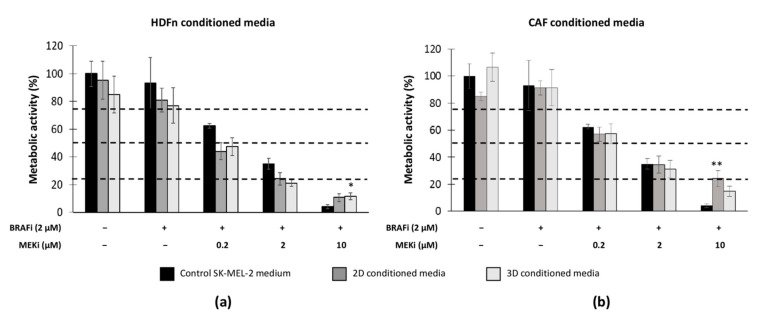
Effect of HDFn- and CAF-conditioned media on 2D SK-MEL-2 sensitivity to BRAFi + MEKi. Conditioned media obtained from (**a**) HDFn or (**b**) CAFs were used for SK-MEL-2 cells cultured as a monolayer and treated with BRAFi + MEKi. SK-MEL-2 cell proliferation relative to the untreated control medium was assessed by cell metabolic activity (MTS assay). Results are means ±  s.d. of three triplicate experiments: * *p* < 0.05; ** *p* < 0.01 indicate significant difference vs. untreated cells with control medium.

To compare the potential effect of the dermal equivalents on the MM cells’ response to a targeted therapy, we investigated SK-MEL-2 proliferation in HDFn or CAF-conditioned media (CM) from 2D and 3D cultures. Briefly, healthy and cancer-associated fibroblasts cultured as a monolayer or within a 3D collagen gel were treated with BRAFi and MEKi for 48 h, and the CM were added to SK-MEL-2 cells cultured in the 2D configuration (Figure 5). Our data revealed that HDFn CM decreased SK-MEL-2 metabolic activity and potentialized the inhibitory effect of BRAFi and MEKi (for 0.2 and 2 µM) (Figure 5a): IC50 were 0.15 µM and 0.17 µM MEKi (+2 µM BRAFi) when MM cells were cultured with 2D and 3D HDFn CM, respectively (the control IC50 of combined therapy on SK-MEL-2 cells was 0.42 µM MEKi + 2 µM BRAFi). For the highest MEKi concentration (10 µM), no beneficial effect of HDFn CM was observed. The CAF CM (2D or 3D) did not modify the sensitivity of SK-MEL-2 cells to the BRAFi and MEKi treatment (Figure 5b), IC50 were approximately 0.4 µM MEKi + 2 µM BRAFi, similarly for the control. However, for 10 µM MEKi, the SK-MEL-2 metabolic activity remained high when the cells where exposed to the CAF CM: IC75 were estimated at 8.8 µM MEKi and 5 µM MEKi (+2 µM BRAFi) in 2D and 3D CM conditions (IC75 control = 4.4 µM MEKi + 2 µM BRAFi) (Table A1).

## 4. Discussion

We previously developed and described a heterotypic 3D melanoma model incorporating stromal cells representing the paracrine signaling and extracellular matrix component [11]. When subjected to vemurafenib (BRAFi), the 3D melanoma coculture model made it possible to identify potent sensitive MM cells. As MM therapy has recently evolved in clinical practice into a combination of targeted therapy compounds (BRAFi + MEKi) [2,3,19], we evaluated the association of vemurafenib and cobimetinib (MEKi) on MM cell lines cultured as a monolayer or cocultured with fibroblasts in a 3D model. We also compared the influence of healthy or melanoma-associated fibroblasts on the MM response to combined therapy.

### 4.1. The Drug Combination Efficiently Inhibits 2D and 3D MM Cell Proliferation Regardless of BRAF Status

We previously reported that BRAFi treatment alone was efficient for inhibiting mutant BRAF V600E MM cell proliferation but had no effect on 2D BRAF wt cells (SK-MEL-2 and HDFn) [11]. Here, the association of vemurafenib and cobimetinib clearly inhibited the cell proliferation for MM cells and fibroblasts, regardless of their BRAF status, with a MEKi dose-dependent effect. The sensitivity of SK-MEL-2 cells to combined targeted therapy indicates that the treatment is interesting in NRAS-mutated malignant tumor cells, including melanoma as previously suggested [20]. Single MEKi treatment significantly inhibited cell metabolic activity for both cell lines but was more efficient in BRAF mutant cells (SK-MEL-28) for low concentrations than in the NRAS mutant. The MEKi concentrations we applied are known to efficiently suppress ERK phosphorylation and activation in SK-MEL-2 cells [20,21]. Our data also indicate that combined BRAF and MEK inhibition was stronger than single MEKi treatment for inhibiting SK-MEL-28 and SK-MEL-2 cell proliferation and that BRAF inhibition was not redundant to MEK inhibition. RAF/MEK pathway inhibition also triggered a significant decrease in cell viability with DNA fragmentation, especially in BRAF wt SK-MEL-2 cells, suggesting that apoptotic cell death was induced. MEK inhibitors were recognized as potent apoptotic inducers in BRAF V600E and NRAS mutant MM cells in a caspase-independent or dependent manner [22,23]. The sensitivity of mutated NRAS MM cells to MEK inhibitor treatment is described increasingly often in the literature, indicating that NRAS gain could be a new therapeutic target for melanoma [24,25,26].

Combined compounds had an inhibitory effect on 3D cell proliferation observed on histological section images and the EdU assay. Cell viability also decreased during the treatment correlated with the increase in TUNEL positive cells. Interestingly, DNA fragmentation was higher in coculture models than in the 2D monocultures, highlighting that the 3D environment plays an important role in treatment effectiveness.

There is very limited cellular analysis of this treatment combination in the literature on coculture models. Recently, a study reported that 3D melanoma cells (mutated BRAF and mutated NRAS melanoma cells) were significantly more sensitive than 2D cells to a combined treatment (dabrafenib and trametinib) [26]. The authors clearly observed that the 3D collagen microenvironment affected how cells respond to treatment and that developing relevant in vitro models is a critical step for efficient drug evaluation.

### 4.2. Healthy or Cancer-Associated Fibroblasts Have a Different Impact on the Sensitivity of MM Cells to Targeted Therapy

Our previous data underlined the significance of paracrine signaling by stromal cells in 3D coculture models for the response of MMs to vemurafenib. We then compared the effect of healthy fibroblasts (HDFn) or MM-associated fibroblasts (CAFs) on the response of SK-MEL-2 to targeted therapy.

CAFs were isolated from a cutaneous MM biopsy and were characterized: cells showed a spindle-shape and flattened morphology, and were strongly positive for vimentin and PDGFRβ according to the literature [27] but were negative for the melanoma marker Melan-A. Isolated CAFs proliferated at a slower rate compared to HDFn, a behavior already described in the literature [28]. The BRAFi + MEKi treatment effectively inhibited the proliferation of CAFs cultured in a monolayer (2D) and within a collagen gel (3D). An inhibitory effect, comparable to that observed with HDFn (3D) cells, was obtained on CAFs cultured within a collagen gel. CAFs acquire the properties of myofibroblasts, reshape the extracellular matrix (ECM) and tissue architecture, and secrete factors that together promote the transformation process by promoting tumor growth, angiogenesis, inflammation, and metastasis, as well as contributing to treatment resistance [13,29]. Several in vitro and in vivo experiments have shown that tumor stroma fibroblasts promote the proliferation of MM cells and that they could be targeted to effectively suppress tumor growth [30,31]. Clearly, a therapeutic combination targeting tumor cells and CAFs could provide promising strategies for improving treatment outcomes and overcoming acquired resistance [32].

**Figure A5:**
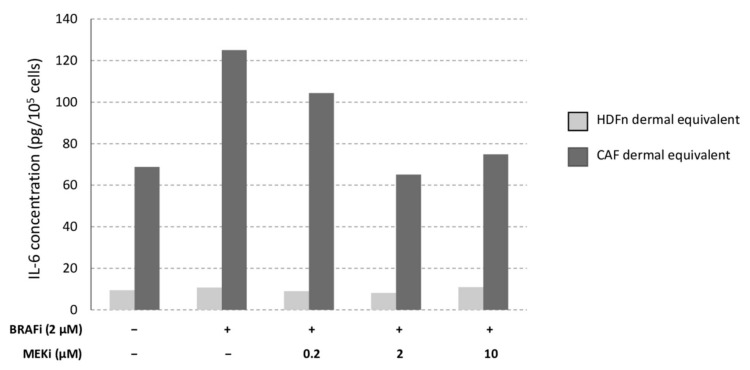
IL-6 release measured in the cell culture media of HDFn and CAF dermal equivalents treated with BRAFi and MEKi for 48 h. IL-6 concentration was quantified by an ELISA assay (Affymetrix eBioscience), *n* = 1.

We also observed that HDFn CM potentialized the efficiency of drugs with the lowest IC50 when MEKi ≤ 2 µM. In contrast, CAF CM did not modify the sensitivity of SK-MEL-2 for the lowest MEKi concentrations but significantly increased the resistance of MM for high concentrations. Together, these results clearly suggest different secretion profiles between healthy and cancer-associated fibroblasts. IL-6 secretion by 2D and 3D HDFn remained low and was not modified during the treatment, whereas treated CAFs secreted up to 120 pg/10^5^ cells of IL-6 (12 times more than HDFn), especially when cells were cultured in the 3D configuration (Figure A5). IL-6 is a well-known pro-inflammatory factor frequently up-regulated in serum and associated with tumor progression [33]. IL-6 is frequently overproduced by CAFs in different types of cancer [34] stimulating cancer cell proliferation, survival, invasiveness, and drug resistance [14,35].

Crosstalk between cancer cells and CAFs is an area that is increasingly under investigation and needs to be taken into account to predict drug efficiency [36,37,38].

## 5. Conclusions

Our data indicate that the combined vemurafenib and cobimetinib treatment efficiently inhibited 2D and 3D MM cell proliferation and survival regardless of their BRAF status. The secretory profiles of fibroblasts influenced the sensitivity of MM to the combined targeted therapy and highlighted the requirement for 3D in vitro cocultures to represent the crosstalk between melanomas and CAFs during preclinical studies of drugs. Further studies will be required to determine the precise differences between CAFs and healthy fibroblast secretomes.

## Figures and Tables

**Table 1:** Percentages of TUNEL positive cells after 48 h of treatment with vemurafenib (BRAFi 2 µM) or vemurafenib + cobimetinib (BRAFi 2 µM + MEKi 2 µM).

Cell Lines	Culture Conditions	No Treatment	BRAFi+/MEKi−	BRAFi+/MEKi+
SK-MEL-28	2D monoculture	0.4% ± 0.4	1.6% ± 1.2	7.0% ± 2.2 ***
	3D coculture	1.3% ± 1.3	2.5% ± 1.7	28.5% ± 10 ***
SK-MEL-2	2D monoculture	4.1% ± 3.4	6.4% ± 3.2 ***	25% ± 8.8 ***
	3D coculture	1.9% ± 1.2	12.8% ± 3.4 ***	17.4% ± 8.6 ***
HDFn	2D monoculture	0.3% ± 0.3	0.5% ± 0.1	6.6% ± 3.2 ***
	3D monoculture	0.5% ± 0.7	1.5% ± 1.5	12.1% ± 9.5 ***

*** *p* < 0.001 indicate significant difference vs. untreated cells.

## Data Availability

The data presented in this study can be made available upon request from the corresponding author.

## Appendix A

**Table A1:** Estimated inhibitory concentrations of MEKi (+2 µM BRAFi) on SK-MEL-2 cells cultured with HDFn or CAF-conditioned media.

Inhibitory Concentrations	SK-MEL-2 Control	HDFn CM		CAF CM	
		2D	3D	2D	3D
IC25 (µM)	0.12	0.04	0.03	0.1	0.1
IC50 (µM)	0.42	0.15	0.17	0.4	0.7
IC75 (µM)	4.4	1.85	1.7	8.8	5
